# 

**Published:** 1890-11

**Authors:** 


### CONTENTS:

Article I.—On the Dangers Arising from Syphilis in the Practice of Dentistry. By L. Duncan Bulkley. - 289
" II.—Dr. Koch's Consumptive Cure. - - - - - 300
" III.—The Preparation and Filling of Roots at One Sitting. By E. L. Clifford, D.D.S. - - - - - 309
" IV.—Deformity of Face Treated with Apparatus. - - 320
" V.—Affections Likely to Occur in the Alveolar Process. - 322

### COMMUNICATIONS.

The California State Board of Dental Examiners, and its Influence on Dental Education. - - - - - - - 324
The Kansas City Academy of Medicine. - - - - - 327

### EDITORIAL.

The Baltimore Dentists and the Census. - - - - - - 327

### MONTHLY SUMMARY.

Primary Anæsthesia in Minor Operations. - - - - - 331

Euphorine. - - - - - - - - - 333

Wandering Rash on Children's Tongues. - - • - - 334

A Local Anæsthetic Formula. - - - - - - - 335

Normal Weight of Man. - - - - - - - - 335

Dangers of Salivation from Calomel and Acids. - - - 336

Cocaine Anæsthesia Obtained by Means of Cataphoresis. - - 336

